# Family-Wide Dysregulation of Phosphodiesterases Alters cAMP/cGMP Microdomains in Thoracic Aortic Aneurysm

**DOI:** 10.3390/jcdd13010023

**Published:** 2026-01-01

**Authors:** Dimitrios E. Magouliotis, Serge Sicouri, Vasiliki Androutsopoulou, Massimo Baudo, Francesco Cabrucci, Prokopis-Andreas Zotos, Andrew Xanthopoulos, Basel Ramlawi

**Affiliations:** 1 Department of Cardiac Surgery Research, Lankenau Institute for Medical Research, Main Line Health, Wynnewood, PA 19096, USA; dimitrios.magouliotis.18@alumni.ucl.ac.uk (D.E.M.); massimo.baudo@icloud.com (M.B.); francesco.cabrucci.6@gmail.com (F.C.); ramlawib@mlhs.org (B.R.); 2 Department of Cardiothoracic Surgery, University of Thessaly, Biopolis, 411 10 Larissa, Greece; androutsopoulouvasiliki@uth.gr (V.A.); zotospro@hotmail.com (P.-A.Z.); 3 Department of Cardiology, University of Thessaly, Biopolis, 411 10 Larissa, Greece; andrewvxanth@gmail.com; 4 Department of Cardiac Surgery, Lankenau Heart Institute, Main Line Health, Wynnewood, PA 19096, USA

**Keywords:** thoracic aortic aneurysm, phosphodiesterase, cyclic nucleotide, PDE10A, cAMP, cGMP, endothelial junctions, vascular remodeling

## Abstract

Background: Thoracic aortic aneurysm (TAA) is driven by complex molecular mechanisms beyond size thresholds, yet the role of cyclic nucleotide metabolism remains unclear. Phosphodiesterases (PDEs), which hydrolyze cAMP and cGMP in compartmentalized microdomains, act as key regulators of vascular integrity and remodeling. Methods: We performed a hypothesis-driven, transcriptomic analysis of 20 PDE isoforms using the GSE26155 dataset (43 TAA vs. 43 controls). Raw microarray data underwent background correction, log2 transformation, and false-discovery adjustment. Differential expression, logistic regression, receiver-operating characteristic (ROC) curves, calibration testing, correlation analysis, and interactome/enrichment mapping were conducted. Results: Thirteen PDE isoforms were significantly dysregulated in TAA. Upregulated transcripts included PDE10A, PDE2A, PDE4B, PDE7A, and PDE8A, whereas PDE1A/B/C, PDE3B, PDE5A, PDE6C, and PDE8B were downregulated. PDE10A achieved excellent discrimination for TAA (AUC = 0.838), while other isoforms demonstrated fair discriminatory ability. Correlation architecture revealed coordinated regulation between PDE subfamilies, including inverse relationships between PDE2A and PDE8B (r = −0.68). Interactome analysis highlighted dense connections with cyclic nucleotide and purinergic signaling hubs, enriched in vascular tone, NO–cGMP–PKG, and junctional assembly pathways. Integrating these findings with epigenetic and junctional frameworks suggests that PDE dysregulation promotes endothelial barrier fragility and maladaptive smooth-muscle remodeling. Conclusions: Family-wide PDE dysregulation characterizes human TAA, with PDE10A emerging as a central transcriptomic signature. Altered cAMP/cGMP microdomain signaling aligns with junctional failure and epigenetic control, supporting the potential of PDE isoforms as biomarkers and therapeutic targets. These results provide experimental evidence that cyclic nucleotide hydrolysis is re-wired in TAA, supporting PDE10A as a novel biomarker and therapeutic target that bridges molecular dysregulation with clinical risk stratification in thoracic aortic disease.

## 1. Introduction

Thoracic aortic aneurysm (TAA) comprises acute and chronic syndromes of the aortic organ, demanding structured surveillance and timely intervention under contemporary guidance. Recent evidence and formal consensus documents have clarified thresholds, imaging pathways, and operative strategies across the spectrum of disease, providing clinicians with a practical compass for decision-making [1,2]. Despite these advances, unmet needs remain for enhanced mechanistic biomarkers that facilitate risk stratification, illuminate disease biology, and propose targeted therapies.

Beyond diameter thresholds and imaging algorithms, converging clinical-biomechanical evidence highlights that tissue material properties strongly influence risk in thoracic aortic disease [3,4]. Two complementary layers of TAA biology have been delineated in recent analyses. First, broad dysregulation of gap junction proteins has been demonstrated in aneurysmal aorta with fair discrimination and good calibration, implicating impaired intercellular communication as a central axis of pathogenesis [5]. Second, a comprehensive synthesis of the epigenetic landscape in thoracic aortic disease highlights how DNA methylation, histone modifications, and microRNAs (miRNAs) coordinate endothelial permeability, smooth-muscle phenotype, and extracellular-matrix (ECM) turnover [2]. These junctional and epigenetic frameworks point toward a deeper regulatory layer that integrates mechanical, inflammatory, and structural cues within the aortic wall. Cyclic nucleotides, particularly cyclic adenosine monophosphate (cAMP) and cyclic guanosine monophosphate (cGMP), act as such integrators by coordinating endothelial cohesion, smooth-muscle phenotype, matrix turnover, and inflammatory tone [2]. Their effects are organized into spatially restricted microdomains shaped by localized synthesis via adenylyl and guanylyl cyclases and precise hydrolysis by PDEs. In this context, PDEs are not merely “housekeeping” enzymes but nodal regulators that determine whether the aortic wall responds adaptively or degeneratively to hemodynamic stress. This conceptual bridge provides a mechanistic rationale for interrogating PDE isoforms in TAA as downstream integrators of the junctional, epigenetic, and biomechanical disturbances already described. Their effects are sculpted by spatial compartmentation via localized synthesis (adenylyl/guanylyl cyclases) and localized hydrolysis by phosphodiesterases (PDEs); downstream effectors include protein kinase A (PKA) and exchange protein directly activated by cAMP (Epac) for cAMP, and protein kinase G (PKG) for cGMP [6,7,8,9,10,11,12].

Despite extensive characterization of structural and biomechanical determinants, the contribution of cyclic-nucleotide microdomain regulation to TAA pathogenesis remains unexplored. Across the vasculature, PDEs terminate cyclic-nucleotide signals and thus act as nodal control points. Several families are especially relevant to aortic biology: Ca^2+^/calmodulin-activated PDE1 isoforms influence smooth-muscle growth and survival; PDE2A integrates cGMP-to-cAMP crosstalk and can suppress protective cAMP signals in specific microdomains; PDE4 family members (including PDE4B) shape inflammatory responses and leukocyte-endothelial interactions; PDE5A and PDE6C preferentially hydrolyze cGMP and therefore intersect with NO-cGMP-PKG vasoprotective pathways; and PDE7/8 fine-tune cAMP in specialized compartments [13,14,15].

Clinical pharmacology highlights the translational stakes. PDE5 inhibitors are widely used; importantly, case reports and forensic series describe temporal associations between PDE5 inhibitor exposure and vascular dissections or hemorrhagic events, observations that do not establish causality but justify careful mechanistic scrutiny in predisposed states [13,14,15]. Complementing these are pharmacovigilance signals indicating disproportional reporting of aneurysm/dissection with PDE5 inhibitors and preclinical data showing that sildenafil aggravated experimental aortic aneurysm, together reinforcing the need to understand isoform- and compartment-specific signaling in aneurysmal tissue [16,17]. Beyond second-messenger enzymes, membrane channel proteins may influence wall biology. A recent transcriptomic analysis implicated aquaporins (AQPs) in TAA, with several isoforms downregulated and showing fair discrimination and calibration, supporting a broader model where transport channels and junctional complexes co-determine aortic integrity [18].

The present study undertakes a hypothesis-driven, family-wide transcriptomic analysis of PDE isoforms in human TAA using a single microarray cohort to minimize batch effects. The findings are integrated with junctional and epigenetic frameworks to propose a unified model in which PDE dysregulation perturbs cAMP/cGMP balance in the precise microdomains that stabilize endothelial junctions and restrain maladaptive smooth-muscle remodeling. Despite extensive investigation into structural, genetic, and biomechanical factors underlying thoracic aortic aneurysm (TAA), the contribution of cyclic-nucleotide signaling remains largely unexplored. This represents a critical translational gap, as compartmentalized cAMP and cGMP gradients tightly regulate vascular tone, endothelial permeability, and extracellular matrix homeostasis [3,4,7]. PDEs, which hydrolyze cAMP and cGMP, orchestrate local signaling “microdomains” that determine whether aortic cells respond adaptively or maladaptively to hemodynamic stress [8,9]. Recent studies in cardiac and pulmonary vascular tissue have demonstrated that PDE compartmentalization is dynamically remodeled during disease progression [10,11,12], yet comparable analyses in human aortic aneurysm tissue are lacking. Given that the aortic wall is constantly subjected to pulsatile strain and oxidative stress, localized PDE expression may serve as a molecular switch between protective remodeling and degenerative dilation [13,14,15].

Therefore, the current study provides a comprehensive, family-wide transcriptomic evaluation of PDE isoforms in TAA, integrating expression, correlation, and enrichment analyses to reveal global network perturbations. This approach complements the prior investigations on aquaporins [17] and gap junction proteins [5], extending the understanding of molecular microdomain remodeling in aortic disease.

## 2. Materials and Methods

### 2.1. Hypothesis

**Figure 1:**
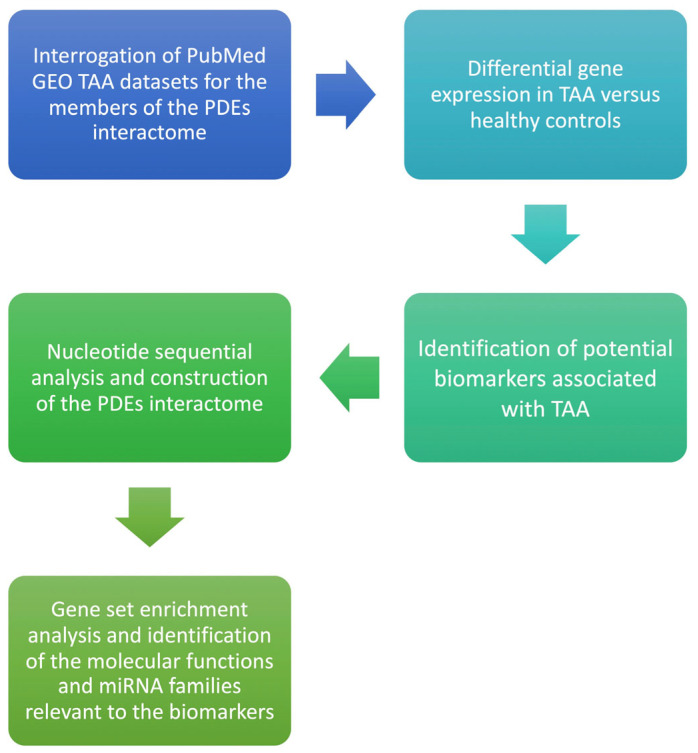
Flow diagram of the bioinformatics analysis for Phosphodiesterase isoform profiling in TAA.

We hypothesized that PDE isoforms are differentially expressed in aneurysmal ascending aorta versus non-dilated control aorta and that such dysregulation confers measurable discriminatory performance for TAA, mapping onto pathways implicated in endothelial permeability, smooth-muscle phenotype switching, and matrix remodeling. The null hypothesis stated no difference in expression between groups. We conducted this work in line with the STREGA recommendations for genetic association reporting [19]. The workflow of the present study is presented in Figure 1. Using a bioinformatics framework, we analyzed publicly available transcriptomic datasets to nominate candidate biomarkers and therapeutic targets for thoracic aortic aneurysm. Such in-silico analyses enable rapid, cost-efficient interrogation of high-dimensional genomic data and can reveal biologically meaningful patterns without collecting new clinical specimens [20]. Central to our workflow was the construction of gene–gene interaction networks to map relationships among differentially expressed genes and to situate them within relevant pathophysiologic pathways. This integrated approach connects complex omics signals to clinical questions and provides a foundation for subsequent mechanistic and experimental validation.

### 2.2. Data Source and Tissue Characteristics

To test the null hypothesis, we drew raw expression data from the NCBI Gene Expression Omnibus (GEO; https://www.ncbi.nlm.nih.gov/gds; accessed 20 June 2025), which archives curated transcriptomic studies. Two investigators (DEM, VA) independently queried GEO with the terms ‘thoracic aortic aneurysm,’ ‘TAA,’ and ‘aortic dilatation,’ limited to Homo sapiens; inter-reviewer agreement was quantified by the kappa statistic. A single eligible microarray dataset was retrieved (GSE26155 [21]) comprising 86 samples (43 TAA, 43 controls). Aneurysmal tissues were sourced from dilated segments (>45 mm), whereas controls were non-dilated donor aortas (<40 mm; mean diameter 34.1 ± 3.6 mm vs. 53.6 ± 7.5 mm in TAA). All specimens had tricuspid valves; 59% exhibited stenosis and 53% regurgitation. No samples with diameters between 40–45 mm were included. Data were generated on the Affymetrix Human Exon 1.0 ST (HuEx-1_0-st) platform. Raw CEL files were processed in R (version 4.5.2) using affy (Bioconductor release for R 4.5) and limma (version 3.66.0) with RMA background correction, log2 transformation, and quantile normalization to ensure comparability. Detailed procedures are described in the Supplementary Material. All analytic scripts and processed data are available upon reasonable request to ensure transparency and reproducibility.

### 2.3. Gene Panel, Preprocessing, and Statistical Testing

A panel of 20 PDE isoforms spanning cAMP- and cGMP-targeting subfamilies was curated a priori. Raw values were log-transformed; distribution normality was evaluated with the D’Agostino–Pearson test. Two-tailed unpaired *t*-tests were used for parametric data and Mann–Whitney U tests for non-parametric data. Multiplicity was controlled by the Benjamini–Krieger–Yekutieli false-discovery approach (target *Q* ≈ 1%).

### 2.4. Discrimination and Calibration

We fitted logistic models per isoform, constructed receiver-operating characteristic (ROC) curves, and calculated AUCs with 95% confidence intervals. Calibration was assessed by observed-to-expected comparisons across deciles and Hosmer–Lemeshow testing (*p* ≤ 0.05 denoting lack of fit) [22,23].

### 2.5. Correlation Structure and Deming Regression

Spearman rank correlations described inter-isoform relationships; significant pairs underwent Deming regression to generate reproducible slope/intercept equations robust to measurement error, enabling cross-dataset testing. Correlations among the differentially expressed genes (DEGs) were assessed using Pearson’s correlation coefficient for data following a normal distribution, and Spearman’s rank correlation for nonparametric comparisons. Deming regression analysis was then applied to quantify the relationship between gene pairs that demonstrated significant associations. Statistical significance was defined as a *p*-value below 0.05, which served as the criterion for rejecting the null hypothesis.

### 2.6. Interactome, Enrichment, and Regulatory Analyses

Protein–protein interaction networks were assembled in STRING and DSigDB [24] at high confidence (score > 0.7). Gene-set enrichment used KEGG/Reactome terms focused on vascular tone, nitric-oxide–cGMP–PKG signaling, and junctional assembly. CpG island mapping was performed at promoter regions; miRNA regulators were predicted through Enrichr/miRTarBase [25]. These CpG-rich regions, typically located within promoter areas, are key epigenetic elements that modulate transcriptional activity through gene silencing mechanisms. Enrichr incorporates data from miRTarBase, a repository comprising over 360,000 experimentally validated miRNA–target interactions [25], facilitating assessment of post-transcriptional regulation. All analyses were finalized in June 2025.

### 2.7. Statistical Rationale

To mitigate type I error inflation across multiple isoforms, the Benjamini–Krieger–Yekutieli procedure was used to control the false discovery rate (FDR) at *Q* ≤ 0.01 [20]. For inter-isoform relationships, correlation coefficients were adjusted using the Benjamini–Hochberg correction. Deming regression was applied for significant gene pairs to account for measurement error in both variables [23]. ROC curves were generated, and AUCs with 95% confidence intervals were compared using DeLong’s nonparametric test [22].

### 2.8. Software

Primary statistics were conducted in GraphPad Prism 10.0.3 (macOS). Networks were visualized with STRING/Cytoscape; schematics were created in BioRender.

## 3. Results

### 3.1. Family-Wide Differential Expression

**Figure 2:**
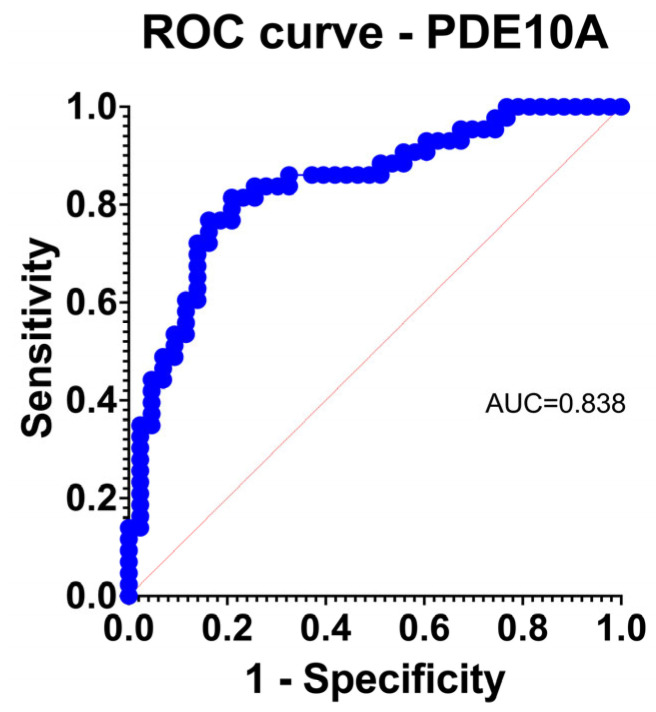
Logistic regression receiver operating characteristic (ROC) curve for PDE10A, demonstrating strong discriminatory power for TAA versus control aortas (AUC = 0.838).

Thirteen of twenty PDE isoforms were differentially expressed between TAA and controls at *p* < 0.05 with concordant false-discovery control. Upregulated isoforms included PDE10A, PDE2A, PDE4B, PDE7A, and PDE8A; downregulated isoforms included PDE1A, PDE1B, PDE1C, PDE3B, PDE5A, PDE6C, and PDE8B. Effect sizes on the log scale were modest but coherent within the family: PDE10A increased by +0.029 (*p* < 0.001), whereas PDE8B decreased by −0.057 (*p* < 0.001). The full statistics are summarized in Table 1 and visualized in Figure 2.

### 3.2. Discrimination and Calibration Properties

PDE10A achieved excellent discrimination for TAA (AUC 0.838) with acceptable calibration by observed-to-expected comparison and Hosmer–Lemeshow testing (Figure 2). Other upregulated genes generally performed in the fair range; downregulated isoforms showed heterogeneous behavior.

### 3.3. Inter-Isoform Correlation Architecture

**Figure 3:**
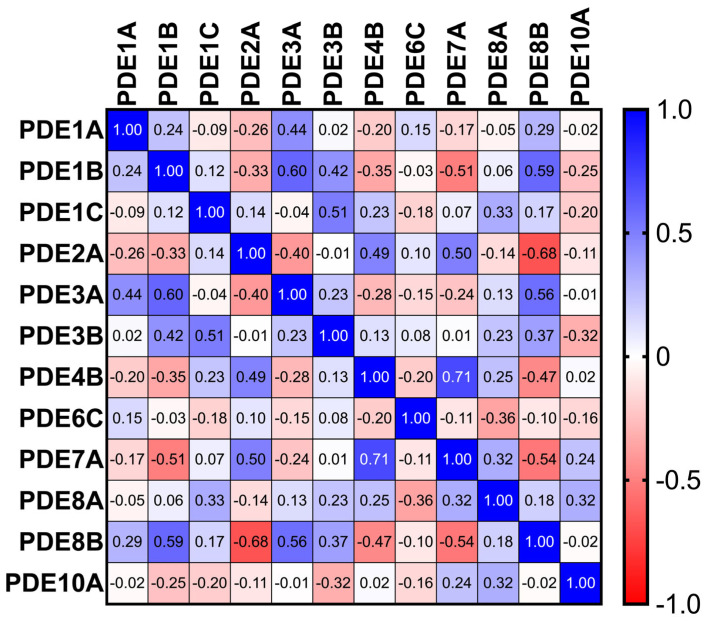
Heatmap of Spearman correlation coefficients among PDE isoforms across TAA samples (*n* = 86), revealing strong co-regulatory patterns among select isoenzymes.

Sixteen significant inter-isoform correlations were identified. Notable positive associations included PDE3A with PDE1B (r = 0.60) and PDE1C with PDE3B (r = 0.51), suggesting coordinated control of cAMP pools; PDE2A correlated inversely with PDE8B (r = −0.68), compatible with competitive or compensatory microdomain regulation. Deming regression yielded robust equations for significant pairs (Figure 3).

### 3.4. Interactome and Enrichment Point to Cyclic-Nucleotide/Purinergic Hubs

**Figure 4:**
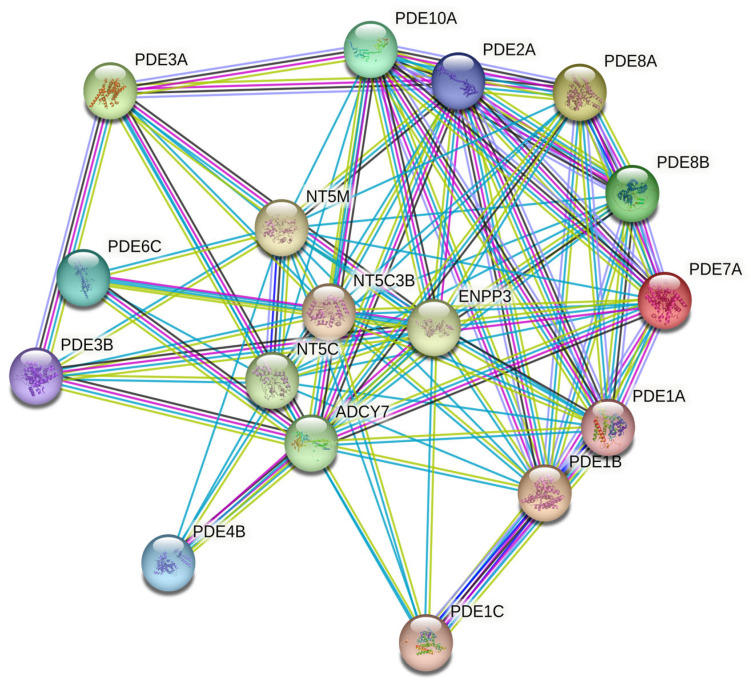
STRING-based protein-protein interaction (PPI) network illustrating known and predicted functional links among PDE isoforms and related nucleotide metabolism genes (confidence score > 0.7).

STRING networks demonstrated dense connectivity among PDEs and partners such as adenylyl/guanylyl cyclases, ectonucleotidases, and transporters, indicating that altered cyclic-nucleotide hydrolysis is embedded within broader nucleotide generation and extracellular metabolism (Figure 4). Gene Set Enrichment Analysis (GSEA) was performed to investigate the functional implications of the DEGs. The top five significantly enriched gene ontologies (GO) terms related to biological processes, together with their corresponding regulatory microRNAs, are presented in Table 2. The predominant biological processes associated with the PDEs encompassed nucleotide catabolic processes, along with vascular smooth-muscle contraction, NO–cGMP–PKG signaling, and junctional assembly pathways. Furthermore, GSEA identified five microRNA families—hsa-miR-330-5p, hsa-miR-6824-3p, hsa-miR-6764-3p, hsa-miR-518c-5p, and hsa-miR-4677-5p—as putative upstream regulators of these differentially expressed PDEs.

### 3.5. Regulatory Programs

**Figure 5:**
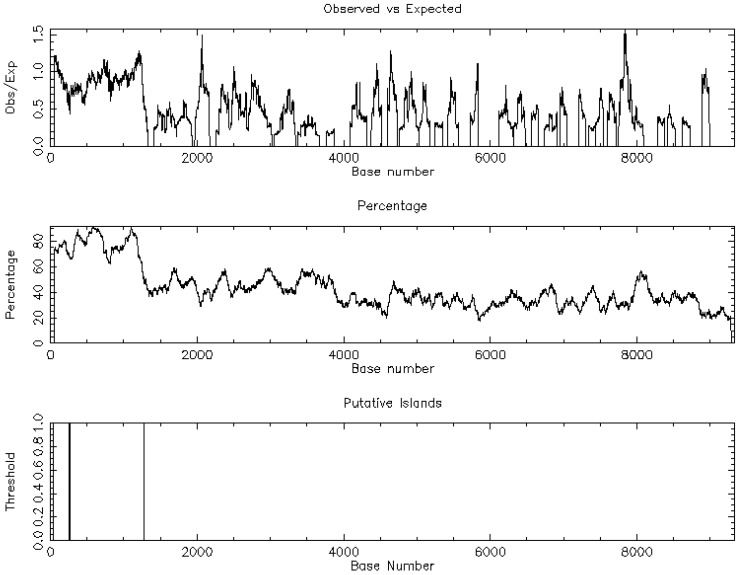
Demonstration of the CpG islands regarding Phosphodiesterase 10A (PDE10A).

Promoter-proximal CpG islands were sparse among top dysregulated isoforms, arguing against island-centric methylation as a dominant driver. Figure 5 demonstrates the one CpG island of PDE10A. In contrast, predicted miRNA families with high affinity for the differentially expressed genes suggested post-transcriptional control in keeping with the wider epigenetic model of TAA [6].

### 3.6. Mechanistic Synthesis with Junctional and Epigenetic Biology

**Figure 6:**
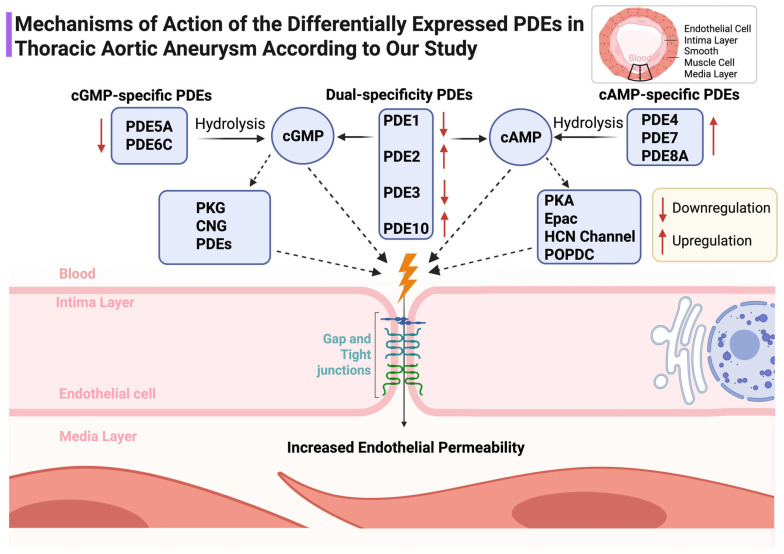
Schematic representation of the mechanistic roles of PDE subtypes in modulating cyclic nucleotide signaling and endothelial barrier integrity in the aortic wall. Upregulation and downregulation in TAA are indicated. ↑: Overexpression ; ↓: Underexpression.

The PDE signature is directionally consistent with heightened endothelial permeability and altered smooth-muscle tone. Downregulation of cGMP-preferring PDE5A and PDE6C alongside upregulation of cAMP-targeting PDE4B, PDE7A, PDE8A and the dual-specific PDE10A would be expected to tilt microdomain signaling toward barrier fragility and maladaptive remodeling. This integrates naturally with our demonstration of junctional protein loss in TAA [5] and with the epigenetic programs summarized previously [6] (Figure 6).

## 4. Discussion

### 4.1. Mechanistic Insights

This family-level interrogation of Phosphodiesterases in TAA reveals a coherent signature in which five isoforms are upregulated (PDE10A, PDE2A, PDE4B, PDE7A, PDE8A) while eight are downregulated (PDE1A/B/C, PDE3B, PDE5A, PDE6C, PDE8B). The prominence of PDE10A, which achieved excellent discrimination, suggests that cyclic-nucleotide hydrolysis is re-wired at the microdomain level in ways that favor endothelial barrier instability and maladaptive smooth-muscle behavior. This interpretation aligns with clinical observations and signals around PDE5 inhibitor exposure and aortic events and supports the premise that isoform-specific and context-specific interventions will be necessary to avoid off-target vascular risk [13,14,15,16,17]. The PDE pattern we observed is compatible with a microdomain-level shift in cyclic-nucleotide signaling that favors endothelial barrier instability and maladaptive smooth-muscle remodeling. PDE4B, PDE7A, and PDE8A are enriched in AKAP-anchored cAMP microdomains that govern PKA/Epac balance and junctional dynamics; their upregulation would be expected to accelerate cAMP hydrolysis in barrier-protective compartments, loosening endothelial cohesion and facilitating leukocyte–endothelial interactions. Upregulated PDE2A amplifies this effect by converting cGMP elevations into reduced cAMP in specific microdomains, thereby weakening cGMP-dependent reinforcement of junctional maturation. In parallel, downregulation of cGMP-preferring PDE5A and PDE6C may disrupt the fine-tuning of NO–cGMP–PKG signaling that stabilizes the cytoskeleton and limits smooth-muscle dedifferentiation. Taken together, these coordinated changes provide a mechanistic link between PDE dysregulation, endothelial permeability, and the smooth-muscle phenotype switching that underlies medial degeneration in TAA.

Mechanistically, cyclic nucleotides regulate vascular integrity through PKA/Epac-dependent cAMP signaling and PKG-dependent cGMP signaling. Endothelial barrier protection is tightly coupled to compartmentalized cAMP in microdomains anchored by AKAP scaffolds; subtle increases in membrane-proximal cAMP can tighten the barrier, whereas cytosolic pools may have divergent effects [7,8,9,10,11,12]. The upregulation of cAMP-directed isoforms (PDE4B, PDE7A, PDE8A) together with downregulation of cGMP-preferring isoforms (PDE5A, PDE6C) supports a scenario in which protective cAMP and cGMP signals are siphoned away from junctional microdomains, weakening endothelial cohesion. PDE2A, that was found upregulated in our data, mediates cGMP-to-cAMP negative crosstalk; increased PDE2A can reduce protective cAMP signals in defined compartments despite elevated cGMP elsewhere, a phenomenon directly visualized with FRET biosensors and extended to vascular junctional maturation [26,27].

Among the upregulated isoforms, PDE4B stands out for its established role in leukocyte activation, microvascular obstruction, and endothelial-to-mesenchymal transition; selective modulation restrains inflammatory injury and EndMT in cardiovascular models [28,29,30]. Conversely, PDE1 isoforms (Ca^2+^/calmodulin-activated) were downregulated. Although PDE1A activity can promote smooth-muscle growth and adventitial myofibroblast activation, reduced transcript levels in bulk tissue may reflect compensatory remodeling or shifts in cellular composition; single-cell and spatial profiling will be required to resolve this [31,32,33].

### 4.2. Biomarker Potential and Clinical Integration

The translational traction of PDE10A merits emphasis. Vascular work demonstrates that PDE10A promotes smooth-muscle proliferation and intimal hyperplasia, partly by antagonizing C-type natriuretic peptide (CNP)/NPR2/cGMP/PKG1α signaling; genetic or pharmacologic suppression restores PKG tone and constrains remodeling [18,27]. If corroborated at the protein level and in circulation (e.g., extracellular vesicles), PDE10A could anchor composite molecular scores alongside junctional and epigenetic markers to improve risk stratification. In this broader schema, channel proteins, including aquaporins, may intersect with cyclic-nucleotide and junctional biology; our recent AQP analysis in TAA underscores this integration and supports development of multi-omic panels [17].

From the same translational perspective, the identification of a PDE10A-centered network signature provides several actionable research directions. PDE10A is known to hydrolyze both cAMP and cGMP and has been implicated in vascular tone regulation and smooth muscle proliferation [29,30,31]. Future experimental work should investigate whether the observed transcriptional upregulation corresponds to increased enzymatic activity or altered cyclic-nucleotide compartmentalization within the aortic wall. Immunohistochemical and proteomic validation, complemented by single-cell or spatial transcriptomic analysis, would clarify the cell-type specificity of PDE10A dysregulation [32,33].

### 4.3. Therapeutic Implications

At the clinical level, quantifying circulating PDE10A fragments or their post-translationally modified forms in plasma or extracellular vesicles may open avenues for minimally invasive biomarker development [34]. Furthermore, selective PDE10A inhibitors, currently under investigation for neurological and metabolic disorders, could be repurposed to test their vascular effects in preclinical aneurysm models [35]. Integrating such molecular readouts with established surgical risk algorithms, such as the European System for Cardiac Operative Risk Evaluation (EuroSCORE) [36], may enable more refined patient stratification and timing of operative intervention. From a translational standpoint, the robust discriminatory performance of PDE10A suggests that it could contribute to composite risk scores rather than functioning as a stand-alone biomarker. A PDE10A-derived molecular index might be integrated with established imaging markers (maximum aortic diameter, growth rate, curvature indices, or longitudinal mechanical metrics) and with serum biomarkers such as D-dimer, matrix metalloproteinases, or circulating TGF-β ligands. In particular, quantification of PDE10A or PDE10A-containing extracellular vesicles in plasma could provide a minimally invasive adjunct for longitudinal surveillance, especially in patients with borderline diameters or discordant biomechanical risk. Such multi-parameter integration aligns with current guideline-directed workflows, in which molecular data refine but do not replace imaging-based decision-making.

### 4.4. Functional Validation

Overall, these findings position cyclic-nucleotide signaling as a missing mechanistic link between biochemical, structural, and clinical determinants of aortic disease. A multidisciplinary framework integrating transcriptomic, epigenetic, and biomechanical datasets could redefine the way aneurysm progression is monitored and therapeutically targeted. To bridge these transcriptomic findings with functional biology, several validation steps are required. Priority experiments include quantitative PDE activity assays in aneurysmal versus control aorta; immunohistochemistry and immunofluorescence to localize PDE10A and PDE2A across intima, media, and adventitia; and proteomic profiling of PDE-interacting partners and downstream effectors within cyclic-nucleotide pathways. Spatial or single-cell transcriptomics could further delineate the cell-type specificity of PDE dysregulation, whereas live-cell FRET-based biosensors for cAMP and cGMP in aortic endothelial and smooth-muscle cells would determine whether microdomain compartmentation is reorganized in TAA. Together, these approaches would clarify whether the transcriptional alterations observed here translate into remodeled cyclic-nucleotide signaling and altered vascular behavior in situ.

### 4.5. Limitations

This study has several limitations. First, reliance on a single GEO microarray cohort introduces inherent constraints, including variability in data quality, heterogeneity of metadata, and potential batch effects. We sought to mitigate these issues by restricting analyses to a single dataset to avoid cross-cohort batch effects, applying uniform preprocessing (RMA, log2 transformation, quantile normalization), and using stringent FDR correction. Nevertheless, validation in independent RNA-seq datasets and multi-center cohorts is needed. Second, transcript levels may not correspond to protein abundance or enzymatic activity, reinforcing the need for functional validation as outlined above. Third, unmeasured confounders, such as PDE inhibitor exposure, antihypertensive or statin use, diabetes, and smoking, may influence PDE expression profiles. Fourth, the cross-sectional design precludes inference about temporal changes during aneurysm progression. Despite these constraints, the coherence of the PDE signature and its mechanistic alignment with junctional, epigenetic, and smooth-muscle biology strengthens the translational relevance of these findings [5,6,7,8,9,10,11,12,26,27,28,29,30,31,32,33].

## 5. Conclusions

Phosphodiesterase dysregulation, particularly the upregulation of PDE10A, emerges as a defining molecular feature of human thoracic aortic aneurysm. The observed transcriptomic profile indicates a shift in cyclic-nucleotide homeostasis that aligns with endothelial junctional failure, vascular smooth muscle dedifferentiation, and epigenetic modulation. Together, these findings delineate a coordinated remodeling of cAMP/cGMP microdomains that may underlie the transition from structural integrity to aneurysmal degeneration. From a translational perspective, the integration of PDE10A expression with junctional and epigenetic biomarkers could enable the development of composite molecular risk scores to complement conventional imaging-based surveillance. Such molecular profiling may improve early detection of patients at risk for accelerated aneurysm growth or rupture, facilitating more personalized timing of surgical intervention. Therapeutically, isoform-specific modulation of cyclic-nucleotide signaling, particularly selective inhibition of PDE10A or context-dependent regulation of PDE4B, represents an emerging strategy to restore vascular homeostasis. As PDE-targeted pharmacotherapy is already established in pulmonary and cardiac disorders, these results provide a strong rationale for repurposing or optimizing PDE inhibitors in the context of aortic disease. Future work integrating transcriptomic, proteomic, and spatial data will be essential to validate these targets and translate molecular insights into clinical application.

## Figures and Tables

**Table 1:** Differential gene expression and significant correlations of the Phosphodiesterase (PDE) genes in Thoracic Aortic Aneurysm.

Gene Symbol	Difference (Actual)	Difference (Hodges-Lehmann)	*p* Values
Downregulated			
PDE1A	−0.012	N/A	0.040
PDE1B	−0.029	N/A	<0.001
PDE1C	−0.034	−0.028	0.002
PDE3A	−0.012	N/A	0.030
PDE3B	−0.038	N/A	<0.001
PDE5A	−0.016	−0.016	0.008
PDE6C	−0.009	N/A	0.020
PDE8B	−0.057	N/A	<0.001
Upregulated			
PDE2A	0.015	0.015	0.026
PDE4B	0.027	N/A	0.007
PDE7A	0.025	0.026	0.001
PDE8A	0.008	N/A	0.042
PDE10A	0.029	0.030	<0.001
Not Significantly Different			
PDE4A	0.004	N/A	0.147
PDE6A	−0.007	N/A	0.055
PDE6B	−0.002	N/A	0.282
PDE6D	−0.004	N/A	0.452
PDE7B	0.005	0.010	0.212
PDE9A	0.007	0.007	0.091

N/A: Not Available.

**Table 2:** Enrichment analysis of gene ontologies (GO) for the Differentially Expressed Genes. The top five relevant biological functions and regulating miRNA families are demonstrated.

Relevant Biological Functions			
	ID	Name	Adjusted *p*-Value
1	GO:1901292	Nucleoside phosphate catabolic process	3.87 × 10^−13^
2	GO:0009166	Nucleotide catabolic process	1.73 × 10^−11^
3	GO:0009187	Cyclic nucleotide metabolic process	2.8 × 10^−11^
4	GO:0009214	Cyclic nucleotide catabolic process	2.8 × 10^−11^
5	GO:0034655	Nucleobase-containing compound catabolic process	2.8 × 10^−11^
Regulating miRNA Families			
	Name		Adjusted *p*-value
1	hsa-miR-330-5p		0.053
2	hsa-miR-6824-3p		0.080
3	hsa-miR-6764-3p		0.080
4	hsa-miR-518c-5p		0.085
5	hsa-miR-4677-5p		0.085

## Data Availability

Supporting raw data is available upon request.

## Supplementary Materials

The following supporting information can be downloaded at: https://www.mdpi.com/article/10.3390/jcdd13010023/s1, STREGA Checklist.
